# Analyzing 24-Hour Blood Pressure Measurements with a Novel Cuffless Pulse Transit Time Device in Clinical Practice—Does the Software for Heartbeat Detection Matter?

**DOI:** 10.3390/diagnostics10060361

**Published:** 2020-05-31

**Authors:** Leo Kilian, Philipp Krisai, Thenral Socrates, Christian Arranto, Otmar Pfister, Andrea Meienberg, Michael Mayr, Annina S. Vischer, Thilo Burkard

**Affiliations:** 1 Medical Outpatient and Hypertension Clinic, ESH Hypertension Centre of Excellence, University Hospital Basel, 4031 Basel, Switzerland; leo.kilian@hin.ch (L.K.); thenral.socrates@usb.ch (T.S.); andrea.meienberg@usb.ch (A.M.); michael.mayr@usb.ch (M.M.); annina.vischer@usb.ch (A.S.V.); 2 Department of Cardiology, University Hospital Basel, 4031 Basel, Switzerland; philipp.krisai@usb.ch (P.K.); otmar.pfister@usb.ch (O.P.); 3 Cardiovascular Research Institute Basel, University Hospital Basel, 4031 Basel, Switzerland; 4 Department of Hematology, University Hospital Basel, 4031 Basel, Switzerland; christian.arranto@luks.ch

**Keywords:** somnotouch, 24-hour blood pressure measurement, pulse transit time, cuffless blood pressure, arterial hypertension

## Abstract

Background: The Somnotouch-Non-Invasive-Blood-Pressure (NIBP) device delivers raw data consisting of electrocardiography and photoplethysmography for estimating blood pressure (BP) over 24 h using pulse-transit-time. The study’s aim was to analyze the impact on 24-hour BP results when processing raw data by two different software solutions delivered with the device. Methods: We used data from 234 participants. The Somnotouch-NIBP measurements were analyzed using the Domino-light and Schiller software and compared. BP values differing >5 mmHg were regarded as relevant and explored for their impact on BP classification (normotension vs. hypertension). Results: Mean (±standard deviation) absolute systolic/diastolic differences for 24-hour mean BP were 1.5 (±1.7)/1.1 (±1.3) mm Hg. Besides awake systolic BP (*p* = 0.022), there were no statistically significant differences in systolic/diastolic 24-hour mean, awake, and asleep BP. Twenty four-hour mean BP agreement (number (%)) between the software solutions within 5, 10, and 15 mmHg were 222 (94.8%), 231 (98.7%), 234 (100%) for systolic and 228 (97.4%), 232 (99.1%), 233 (99.5%) for diastolic measurements, respectively. A BP difference of >5 mmHg was present in 24 (10.3%) participants leading to discordant classification in 4–17%. Conclusion: By comparing the two software solutions, differences in BP are negligible at the population level. However, at the individual level there are, in a minority of cases, differences that lead to different BP classifications, which can influence the therapeutic decision.

## 1. Introduction

Globally, arterial hypertension is one of the most important risk factors for morbidity and mortality [1]. Correct blood pressure (BP) measurement is, therefore, the cornerstone for the diagnosis of arterial hypertension and for therapy monitoring. An overview of current non-invasive blood pressure measurement techniques is summarized in Table 1.

Prerequisites for valid BP measurement include the use of validated devices and validated BP measurement protocols. Since in-office BP measurements are prone to errors, out-of-office techniques such as 24-hour BP measurement are more frequently being implemented. A 24-hour BP measurement using a cuff-based device is currently recognized as the standard for the diagnosis of arterial hypertension [1,2,7]. These devices have been validated for accuracy according to specific validation protocols against the gold-standard BP measurement technique using mercury sphygmomanometers and auscultation. During the last decade, most devices have been validated according to the specifications of the European Society of Hypertension International Protocol Revision 2010 for validation of Blood Pressure Measuring Devices in Adults (ESH IP 2010) [8]. A cuff-based 24-hour blood pressure measurement consists of a predefined number of single measurements and a standardized, guideline-based approach for the calculation of mean 24-hour, awake, and asleep values. This technique is robust and easy to apply in clinical practice [3].

**Figure 1:**
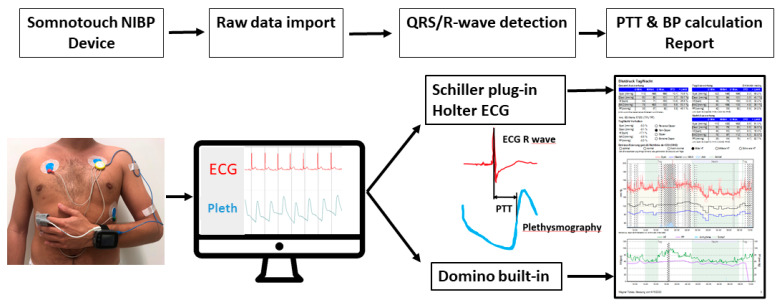
Illustration of the steps which need to be done during the analysis of a Somnotouch-NIBP exam. A patient wearing the device as illustrated. After raw data import, the QRS/R-wave detection is done by the Domino light software, or in the case of Holter ECG analysis by the Schiller plug-in software. From the ECG, QRS/R-waves are detected and used to calculate the pulse transit time. Finally, BP values are calculated according to specific algorithms. BP values are processed and displayed in the form of a diagnostic report. NIBP indicates non-invasive blood pressure; PTT: pulse transit time; BP: blood pressure; ECG: electrocardiography; Pleth: plethysmography.

However, standard cuff-based 24-hour BP measurement devices have limitations in their clinical use. Cuff-based devices do not measure BP continuously, are prone to errors during movements, and interfere with daily activities and sleep [3]. Also, in obese patients, finding the correct cuff size for the patient´s arm can be problematic and may lead to false measurements and discomfort [9]. New devices, which use pulse-transit-time (PTT) measurements for the calculation of BP values, have the potential to overcome these problems. PTT is the time it takes for a pulse wave to travel from the heart to a peripheral organ. This time delay is inversely correlated to BP [4]. The device uses this data in various algorithms and calculates a BP value [4]. The Somnotouch-Non-Invasive-Blood-Pressure (NIBP) (by Somnomedics GmbH, Randersacker, Germany) is a commercially available, CE (Conformité Européenne) marked, PTT-device which assesses BP over a 24-hour period after an initial calibration. Together with the 24-hour BP examination, a Holter electrocardiography (ECG), pulse-oximetry, and actigraphy can be simultaneously recorded and analyzed. For BP, the device has been validated according to ESH IP 2010 over a period of 20–25 min [5,10]. The device works with its own software package (Domino light, by Somnomedics GmbH, Randersacker, Germany) and is additionally compatible with a 24-hour Holter electrocardiography (ECG) analysis software package (Schiller Darwin, by Schiller AG, Baar, Switzerland) for heartbeat (HB) detection. This Schiller Darwin software plug-in adds a semiautomated 24-hour ECG analysis function to the Domino light software and is usually implemented if cardiologists use the Somnotouch-NIBP for additional Holter ECG analysis. As a plug-in software, it has to be purchased additionally. The steps which have to be done during the analysis of a Somnotouch-NIBP examination until the final report are shown in Figure 1. Usually, HB detection is done by Domino light software, but in cases where the physician decides to use Schiller software plug-in for the ECG analysis, the results of the Schiller HB detection are used for PTT calculation. This is the automatic setting when using Schiller software; however, this can be changed back to the Domino light HB detection in the advanced system’s settings. However, it remains unclear to what extent BP values calculated after the use of the Schiller plug-in software differ from the BP values that would have been calculated if only using the integrated Domino light software solution.

In 2019, we published the results of the Somnotouch-NIBP device compared with a reference cuff-based device [6]. Differences were seen in the values obtained by the two methods of analysis. Although not all comparisons were statistically significant, a clinically relevant BP difference at the individual level could not be excluded [6]. The aim of the current study was to analyze the impact and clinical significance of the two different HB detection software programs commonly used with the Somnotouch-NIBP device on 24-hour BP results in a larger cohort.

## 2. Materials and Methods

### 2.1. Study Population

In the present analysis, we used data from a prospective cohort of long-term survivors of allogeneic hematopoietic stem cell transplantation (Cardiovascular Outcome after Allogeneic Stem cell Transplantation Cohort (COAST)), that had transplantation between 2000 and 2016. The COAST cohort is a prospective, interdisciplinary, single-center cohort in close collaboration of the Department of Hematology, Department of Cardiology, and the Medical Outpatient Department and Hypertension Clinic at the University Hospital Basel. In brief, the COAST cohort consists of patients, which were included after giving informed consent during their annual follow-up consultation from April 2015 onwards. Patients with complete hematological remission for ≥1 year were included. Patients <18 years of age or with a relapse of the hematological malignancy were excluded. The COAST study focused on accruing data regarding cardiovascular outcomes in this cohort, and therefore, all participants were required to have a 24-hour blood pressure measurement and Holter ECG in close collaboration with the Hypertension Clinic. Therefore, we used the Somnotouch-NIBP (Somnomedic GmbH, Randersacker, Germany) device as part of the standard study procedure. 

The study protocol complied with the Declaration of Helsinki and was approved by the local ethics committee (Ethikkommission Nordwest- und Zentralschweiz (EKNZ 2014-376). Anonymized data supporting the findings of this study are available from the corresponding author upon reasonable request.

### 2.2. Device Characteristics and Placement

Device details were previously described [4,5]. The Somnotouch-NIBP system estimates BP using the PTT technique. The Somnotouch-NIPB is cuffless and allows for continuous beat-to-beat BP monitoring. The system consists of the following parts: a wrist-worn device with an integrated actigraph, a finger photoplethysmography, and three ECG leads (Figure 1). The system measures the transit time of a pulse wave from a HB in the ECG (R-Wave) to the finger plethysmography signal. After calibration with a single, standard cuff-based calibration measurement on the contralateral arm, the Somnotouch-NIBP system calculates a BP value for each PTT according to a predefined algorithm [6]. In brief, levels for systolic and diastolic values are calculated with a non-linear model incorporating changes of the PTT and its relation to BP [6]. A shorter PTT reflects a faster pulse wave propagation and is, thus associated with higher BP values, while lower BP values are associated with slower pulse wave propagation and accordingly, longer PTT [6,11,12].

The device has been in use in our study center since April 2015. In-house training of the device and its calibration procedure took place over a two weeks period prior to initiation of enrollment of the COAST cohort. A standard operating procedure for the device according to the operating manual provided by the manufacturer was implemented.

Patients were educated on the correct handling of the device. The device was then mounted on the participant in a sitting, upright position with legs uncrossed and back supported. The left arm was used for the wrist-worn device and the left index finger for the photoplethysmograph. The right arm was used for the calibration measurement, using an appropriately sized cuff, with an Omron-HBP-1300 device [13,14]. The calibration measurement was taken after 5 min of rest. The value was entered directly into the Somnotouch-NIBP device. Then the calibration device was removed. 

### 2.3. Assessment of 24-Hour Blood Pressure Measurement Results

After completion of the 24-hour BP period, the data were analyzed using both software packages (Figure 1). Samples with less than 20 hours of recording were excluded from further analysis. 24-hour ECGs were analyzed and corrected according to local standards using the Schiller Darwin 24-hour ECG software package (Medilog Darwin V2.5.2.52, by Schiller AG, Baar, Switzerland). The Schiller software results were then used for the calculation of the 24-hour mean, awake and asleep values of systolic and diastolic BP (SchillerBP). After evaluating ECG leads I, II, and III, a board-certified cardiologist selected the lead with best signal quality, and the raw data was subsequently reanalyzed with the Domino Light Software package (Domino light V1.4.0; Somnomedics GmbH, Randersacker, Germany) using the lead with best signal quality. The values thus obtained were used to calculate the 24-hour mean, awake and asleep values of systolic and diastolic BP (DominoBP). To evaluate the possible influences of ECG characteristics on the calculated BP results, ECG tracings were checked for left or right bundle branch blocks, persistent or paroxysmal atrial fibrillation, and the burden of premature atrial contractions (PAC) and premature ventricular contractions (PVC).

### 2.4. Blood Pressure Classification

Calibration measurements were classified according to ESH IP 2010 protocol: low (<130 mm Hg), medium (130–160 mmHg) and high (>160 mmHg), for systolic blood pressure [8]. For diastolic BP the categories were: low (<80 mmHg), medium (80–100 mmHg) and high (>100 mmHg) [8]. Additional categories were used for the final results of the DominoBP, and SchillerBP mean awake BP values according to the guidelines of the National Institute for Health and Care Excellence (NICE) definition for ambulatory BP measurement: Stage 1 hypertension ≥ 135/85 mmHg, stage 2 hypertension ≥ 150/95 mmHg, and severe hypertension (measured in a clinical setting) ≥ 180/110 mmHg [15].

### 2.5. Statistical Analysis

Numeric data are presented as mean ±standard deviation (SD) and median (interquartile range (IQR)) as appropriate. Awake BP for SchillerBP and DominoBP were classified according to the aforementioned NICE definition [15]. BP results were checked for equal distribution using Kolmogorov–Smirnow and Shapiro–Wilk-Test. Bland-Altmann plots were constructed to compare the mean difference between SchillerBP and DominoBP, according to the ESH IP 2010 [8]. We derived the bias and limits of agreement (bias ± 2 SD) from Bland-Altman analysis. Mean differences (SD) were calculated (SchillerBP-DominoBP). Additionally, the means of the absolute differences between DominoBP and SchillerBP with their corresponding standard deviations (SD) were calculated. Correlation coefficients for the results of the two software applications were calculated, and scatter plots were constructed. To check for linear regression and systematic error, residual plots were constructed. Mean BP values were compared using Wilcoxon signed-rank test. Accuracy tables, adapted from the ESH IP 2010 protocol, were constructed [8]. A BP difference of > 5 mmHg between the results generated by DominoBP and SchillerBP software was deemed as clinically relevant and further analyzed. In samples with a BP difference of > 5 mmHg in at least one calculated data (overall six datasets: systolic and diastolic 24-h mean-, awake- and asleep-BP values) the values generated by the DominoBP and SchillerBP software were grouped according to the NICE hypertension definition, and the percentage of concordance and discordance between the two software packages was visualized in four-quadrant matrices. Group comparisons for ECG Characteristics were made using the Fisher’s exact test.

All statistical analyzes were done using SPSS 22/25 (SPSS Inc, Chicago, IL, USA); accuracy tables were done with Microsoft Excel 2016 (Microsoft, Albuquerque, NM, USA). Two-sided *p*-values < 0.05 were regarded statistically significant.

## 3. Results

### 3.1. Baseline Characteristics

In total, 274 BP measurements from 274 patients recruited from April 2015 until November 2017 were reviewed for eligibility. Of these, 40 (15%) measurements had less than 20 hours of recording time and were, therefore, excluded, leaving 234 measurements for the present analysis. Baseline characteristics are shown in Table 2. The mean age was 51.9 years (range, 19–75 years) and 63.2% were male. The calibration measurements showed a systolic mean (standard deviation, (range)) of 129 mmHg (± 23, (81–223 mmHg)) and a diastolic mean of 83 mmHg (± 16, (55–116 mmHg)). The distribution of the results for the calibration measurement according to the ESH-IP 2010 and, for the DominoBP and SchillerBP awake measurements, according to the NICE grading are shown in Table 2 [8,15].

Electrocardiographic characteristics of the whole cohort as well as patients with a difference of > 5 mmHg between DominoBP and SchillerBP are presented in Table 3. There was a low number of patients with persistent or paroxysmal atrial fibrillation, PAC, or PVC burden > 5%, or bundle branch blocks within our cohort. 

### 3.2. Comparison of Blood Pressure Results Derived from DominoBP and SchillerBP Software

Comparisons of mean systolic and diastolic values of DominoBP vs. SchillerBP are shown in Table 4. Median (IQR) DominoBP systolic awake values (130 (119–143) mmHg) were significantly lower than SchillerBP (131 (119–144) mmHg) (*p* = 0.022). Systolic 24-hour and asleep BP measurements, as well as 24-hour, awake, and asleep diastolic BP values, comparing the DominoBP and SchillerBP software showed no significant differences (Table 4).

Mean BP differences and mean absolute BP differences between systolic and diastolic BP values generated by the DominoBP and SchillerBP software are shown in Table 5. 

Mean difference (SchillerBP – DominoBP) of systolic 24-hour BP values (SD) was 0.22 mmHg (± 2.59) (Table 4 and Table 5). The corresponding mean absolute difference (SD) was 1.5 mmHg (± 1.7) (Table 5). Overall, the agreements of both software solutions within ≤2 mmHg, ≤5 mmHg, ≤10 mmHg, and ≤15 mmHg were 81.1%, 94.8%, 98.7%, and 100% for systolic and 87.6%, 97.4%, 99.1%, and 99.5% for diastolic 24-hour BP values, respectively (Table 6 and Table 7). The distribution of agreements over BP categories for systolic and diastolic BP results are shown in Table 6 and Table 7.

**Figure 2:**
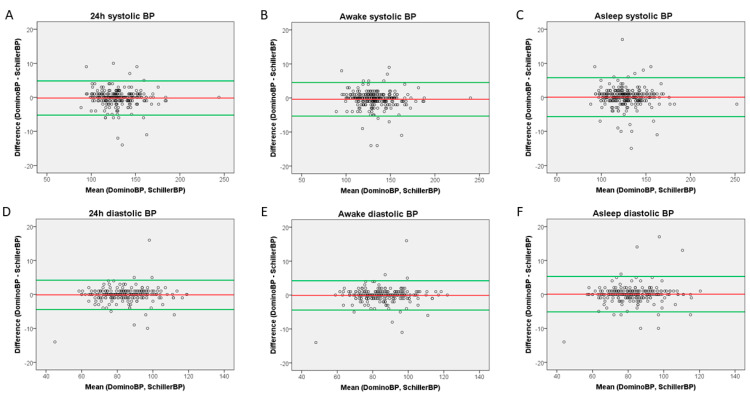
Bland–Altman Plots. (**A**) 24-hour systolic measurements, (**B**) awake systolic measurements, (**C**) asleep systolic measurements, (**D**) 24-hour diastolic measurements, (**E**) awake diastolic measurements, (**F**) asleep diastolic measurements.

Bland–Altman plots comparing BP results generated by DominoBP and SchillerBP software are shown in Figure 2. There were no systematic differences, but several outliers (Figure 2). 

**Figure 3:**
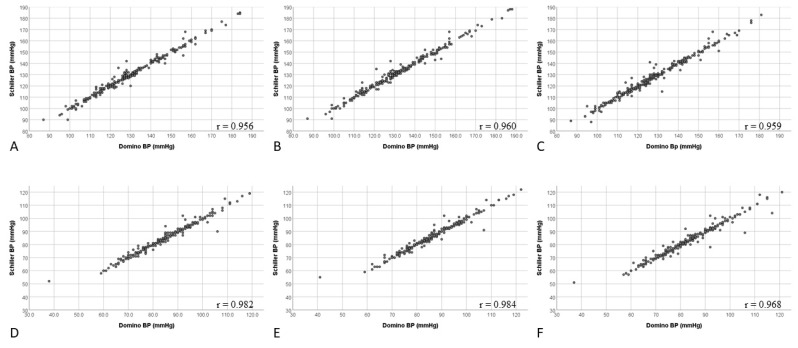
Correlation of systolic and diastolic blood pressure values generated by DominoBP and SchillerBP software for 24-hour, awake, and asleep blood pressure measurements. (**A**) 24-hour systolic measurements, (**B**) awake systolic measurements, (**C**) asleep systolic measurements, (**D**) 24-hour diastolic measurements, (**E**) awake diastolic measurements, (**F**) asleep diastolic measurements.

Correlation coefficients for BP results generated by DominoBP and SchillerBP software were 0.95, 0.96, and 0.95 for systolic and 0.98, 0.98, and 0.96 for diastolic 24-hour-, awake-, and asleep-BP values, respectively (Figure 3).

**Figure 4:**
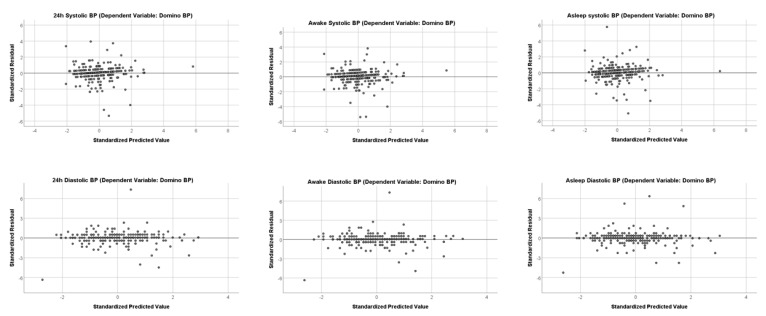
Residual Plots. Independent Variable is SchillerBP for all Residual Plot.

Residual plots are shown in Figure 4; they show unbiased and homoscedastic patterns with few outliers for both systolic and diastolic values during the different measurement periods.

### 3.3. Analysis of Samples with an Absolute Blood Pressure Difference of >5 mmHg between Data Generated by DominoBP and SchillerBP Software 

Baseline characteristics of patients with a BP difference of >5 mmHg in at least one calculated data set (systolic or diastolic 24-h, awake, or asleep BP measurement) between data generated by DominoBP and SchillerBP software can be seen in Table 8. ECG characteristics were presented in Table 3. There was a low number of patients with PVC burden >5% or bundle branch blocks and no statistically significant differences between the groups.

Differences of >5 mmHg were found in a total of 24 (10.3%) patients. The relative distribution of BP differing > 5 mmHg across BP categories is shown in Table 8.

These 24 (10.3%) patients generated a total of 144 BP data sets (24 × 6 datasets consisting of 24-hour-, awake-, and asleep- values for both systolic and diastolic BP). An absolute BP difference > 5 mmHg was found in 41% (*n* = 59/144) of the data sets. Based on the total study population (*n* = 234) with a total of 1,404 data sets, there was a BP difference > 5 mmHg in 4% (*n* = 59/1404) of the data sets. 

### 3.4. Impact of an Absolute Blood Pressure Difference of > 5 mmHg between Data Generated by DominoBP and SchillerBP Software on Blood Pressure Classification

**Figure 5:**
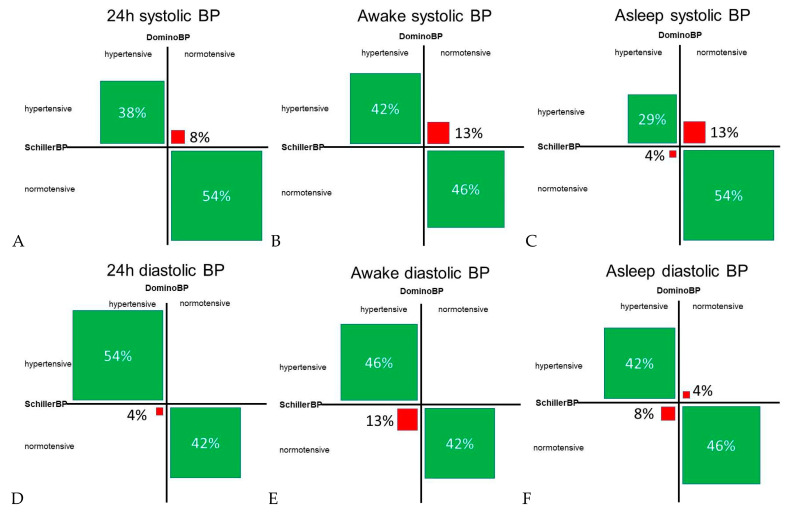
Concordant and discordant blood pressure classification in cases with a systolic and diastolic blood pressure difference of > 5 mmHg between blood pressure data generated by DominoBP and SchillerBP software. Normotensive values for systolic BP: (**A**) BP < 130 mmHg, (**B**) BP < 135 mmHg, (**C**) BP < 120 mmHg; normotensive values for diastolic BP: (**D**) BP < 80 mmHg, (**E**) BP < 85 mmHg, (**F**) BP < 70 mmHg; hypertensive BP is defined as ≥ the defined normotensive BP values; green fields: BP classification concordance between data generated by DominoBP and SchillerBP, i.e., both generated are classified as hypertensive or normotensive BP; red fields: BP classification discordance between data generated by DominoBP and SchillerBP, i.e., according to DominoBP generated data BP values are classified as hypertensive BP, while according to SchillerBP, thegenerated data BP values are classified as normotensive BP and vice versa); BP: blood pressure.

Regarding the classification of normotensive versus hypertensive in subjects with a BP difference of > 5 mmHg between data generated by DominoBP and SchillerBP software, we found discordance in BP classification in 4% to 17%, depending on systolic or diastolic BP and the analyzed time period (24-hour, awake, and asleep BP) (Figure 5). BP values calculated by DominoBP compared to the SchillerBP software resulted in a numerically more normotensive systolic and in less normotensive diastolic BP values (Figure 5).

## 4. Discussion

The Somnotouch-NIBP device is a 24-hour BP measurement device using a combination of new technology and classic diagnostic tools to calculate BP. Another novelty of this system is the possibility to acquire a Holter ECG, pulse oximetry, and actigraphy simultaneously. However, the benefits of this device need to be objectively interpreted in clinical practice. The device’s processing of raw data and beat-to-beat BP calculation is more complex than the calculation of mean values out of standard cuff-based BP measurements. BP results of the Somnotouch-NIBP device can be generated with two different software packages processing the same raw data, Domino light, and Schiller. As both software programs come with the device and the Schiller software plug-in is mainly used for additional Holter ECG analysis, the clinician is likely to assume that the results are interchangeable. This assumed lack of difference is inadvertently reiterated due to the fact that the manufacturers do not bring this to the attention of the user. To date, the present study is the largest analysis of BP results generated with these two software programs, derived out of a prospective cohort, where the Somnotouch-NIBP was used as part of the routine study procedure. 

We found no significant differences in the mean systolic and diastolic BP values between the two groups except for mean systolic awake BP values, where the mean absolute difference was 1.5 mmHg, which was clinically significant. On the other hand, our results show that with the same sets of raw data, the different software solutions do not deliver systolic and diastolic results within 2 mmHg of agreement in approximately 20% and 12% of the patients, respectively. In addition, approximately 10% (24/234) of all patients showed a difference of > 5 mmHg in at least one calculated data set when comparing DominoBP and SchillerBP. In these patients, the calculated DominoBP leads to less systolic but more diastolic hypertensive values compared to SchillerBP, along with a discordant BP classification in 4% to 17%, depending on the analyzed data set.

Therefore, our study elucidates that the same raw dataset, analyzed with two different software packages, leads to two different results.

Importantly, physicians and especially cardiologists have to be aware that with the decision, e.g., to use the Schiller plug-in, there is the possibility that higher BP values may be reported, with the risk of different treatment decisions. 

However, in the current study, the disagreement between DominoBP and SchillerBP software derived BP values were less pronounced than in our previous study, which revealed an absolute mean difference in mean systolic and diastolic 24-hour blood pressure of ≥5 mmHg in 12.5% and 4.2% of cases, respectively [6]. This is can partially be attributed to the smaller data set in our previous study, which led to an overestimation of the disagreement in the calculation between the two software packages [6]. An additional factor may be the difference between the two patient populations with younger and healthier participants in the previous study. This may have led to more daytime activity and motion artifacts in the ECG. This may have been detected differently by the software. Unfortunately, in other studies comparing Somnotouch-NIBP to cuff-based blood pressure measurements, only one type of data processing was used, and information about the differences between Schiller Darwin and Domino software is lacking [5].

Our results show that in subjects with BP values on the threshold for starting therapy, a closer look should be taken to see whether their results were generated with Domino or Schiller Darwin software. In these cases, it would be worthwhile to calculate the data with both software packages, as not to miss outliers who are on the border of having a clinically relevant diagnosis.

Another question that remains to be clarified is why these two software programs calculate different results. With an overall negligible mean difference and high correlation coefficients between DominoBP and SchillerBP, there seems to be no systematic error between the two software variants. As both software use the same raw data, intra- or interindividual aspects, which may result in higher or lower BP values seem unlikely. The PTT method calculates BP values based on changes in the PTT after a single cuff-based calibration BP measurement at the beginning of the 24-hour measurement period. For a reliable and stable calculation of the PTT, robust and uniform detection of a specific time point of the QRS complex and the photoplethysmographic signal is crucial [10]. Since raw data from the photoplethysmographic signal used for the PTT calculation are the same, there must be a difference between the Domino and Schiller Darwin software algorithm for HB detection, the latter of which was originally developed for 24-hour ECG analysis and automated arrhythmia detection. Since the PTT is measured in milliseconds, even minor changes from R-Wave to S-Wave in the HB detection may lead to relevant changes in the calculated PTT, and therefore, to changes in BP measurement results [10]. One could assume that the programming of Schiller Darwin Software may be less precise for a stable HB detection in the context of PTT calculation as it was originally developed for a different purpose.

### 4.1. Limitations

For the present analysis, we used data from a prospective cohort of long-term survivors after hematopoietic stem cell transplantation. Although this seems to be a special patient group because of possible comorbidities or concomitant medication, which may influence the vascular function, the cardiac rhythm and the ECG, e.g., QRS complex or QTc interval, we chose this cohort because, to date, this is the largest cohort of prospective, high-quality Somnotouch-NIBP measurements. Our study focuses on one part of raw data processing, specifically the detection of a QRS-complex or R-wave, which is not interfered with by vascular properties or other QTc intervals. There is the possibility that bundle branch blocks or, e.g., PVC theoretically interfere with a stable HB detection and BP estimation by the two systems but there are no previous Somnotouch-NIBP studies addressing these questions, and there were no restrictions for the use of the Somnotouch-NIBP device in case of conduction abnormalities or cardiac arrhythmias formulated by the manufacturer. Additionally, we found a very low prevalence of left or right bundle branch blocks, atrial fibrillation, PAC, or PVC in our cohort. Therefore, we think that the results in our cohort are not biased by disease-related ECG abnormalities.

There was no parallel, cuff-based gold standard 24-hour BP measurements, so we cannot answer the question of which of the two software packages is best comparable to this gold standard. 

We defined the ECG-lead for the calculation of Domino-BP after a visual check by an experienced, board-certified cardiologist for ECG quality and signal stability, yet there may be a user-specific bias. This approach is standard in our institution to reduce ECG artifacts. Since there is an initial calibration of the PTT to a standard-cuff based blood pressure measurement, results should not be influenced.

As both software algorithms are not transparent for the end-user, it was not foreseeable which difference in signal processing leads to the differences in BP values. Finally, the current results only apply to the software versions examined. In the case of software updates, new analyzes will be needed.

### 4.2. Perspectives

With the Somnotouch-NIBP device, there exists a device that may overcome some of the limitations of a standard cuff-based BP 24-hour measurement. It gives the physician a beat-to-beat picture of the individual BP profile of the patient, which delivers more information when compared to intermittent measurements. The device is clinically validated according to the recommended protocol for single measurements over a short-term period [5]. In clinical use over 24 h, it has to be kept in mind by physicians that the values obtained by the device are not directly interchangeable and higher, especially at night compared to usual cuff-based devices [6,16]. Therefor more evidence is needed to guide diagnostic and treatment decisions. The best way to generate this evidence would be large epidemiological studies associating BP values of the device to clinical outcomes but such studies are resource-intensive. An alternative method would be to define device-specific cut-off points that correlate to established standard cuff-based 24-hour devices in larger cohorts with various patients. Furthermore, and based on our results, there should be a single, easy, and standard procedure for raw data processing provided by the manufacturer. This will result in higher quality, which is valuable not only in clinical practice but also in clinical trials. Until then, we recommend that BP analysis with the Somnotouch-NIBP device should be done with the Domino light software since this is available to all customers.

## 5. Conclusions

When comparing DominoBP and SchillerBP software for calculating 24-hour BP measurements based on the PTT method in Somnotouch-NIBP, the differences in blood pressure results at the population level are negligible. However, at the individual level, in the minority of cases, there are also differences that lead to a different BP classification and thus can influence the therapeutic decision. Therefore, our data underline that when a new device is introduced into the market, all different applications and ways of data processing have to be thoroughly studied and validated. 

## Figures and Tables

**Table 1:** Overview of different non-invasive methods for office and out-of-office blood pressure measurement.

Method	Device/Components	Basic Principles of Measurement	Advantages	Disadvantages
Mercury Sphygmomanometer	Sphygmomanometer and auscultation by observer	Manual inflation and deflation of the cuff and auscultation of Korotkoff sounds. First sound appearing is the systolic blood pressure, the complete disappearance of the sound indicates diastolic blood pressure	Gold-standard for non-invasive brachial blood pressure measurement. Reference device in validation studies for single measurement comparisons	Safety and economic concerns related to mercury use. Prone to observer related errors. No ABPM * possible
Aneroid Sphygmomanometer	Sphygmomanometer and auscultation by observer	Lever and belly system instead of mercury sphygmomanometer	No mercury. Low-priced devices	Prone to observer related errors. No ABPM possible
Oscillometric or auscultatory automated BP devices	Monitor including cuff and bladder (Different validated models commercially available)	Oscillometric pressure changes of cuff pressure or Korotkoff sounds are registered by electronic sensors in the cuff, and BP is calculated by device-specific algorithms ABPM by single intermittent measurements over 24 h, usually every 20 to 30 min	Cost-effective. Standard in most clinical settings. Specific devices for ABPM available. Devices can be used for at-home blood pressure measurement.	Different measurement protocols available. Large number of non-validated devices on the market. Discomfort for the patient. Intermittent measurement in case of ABPM.
Pulse-Transit Time Measurement (PTT)	Monitor, Finger-photoplethysmograph, ECG (e.g., Somnotouch-NIBP (Somnomedic GmbH, Randersacker, Germany))	Time-interval between R-wave on the ECG and the arrival of the corresponding pulse wave at the finger-photoplethysmograph (PTT) can be calculated. After calibration to a single standard, BP measurement changes in PTT are translated into changes of BP values according to specific algorithms. Beat-to-beat BP calculation.	Less discomfort for patients. Device for ABPM. Beat-to-beat blood pressure measurement possible. 24h-ECG, pulse oximetry and actigraphy as additional information over 24 h.	Validated for single measurements. Seems to result in higher BP values, when used over 24 h compared to standard devices. More complex analysis of examinations.
Volume-clamp-technique Finapres (FINger Arterial PRESsure)	Finger-cuff and bladder, Finger-plethysmograph (Finapres (Finapres Medical Systems BV, Enschede, Netherlands))	Finger arterial pressure and waveform is measured using a finger cuff and an inflatable bladder in combination with a finger-plethysmograph. After calibration to standard BP, beat-to-beat blood pressure is calculated according to specific algorithms.	Less discomfort for the patient. Beat-to-beat blood pressure measurement.	Incomplete validation. No ABPM possible. (Portapress not available on the market anymore) Used mainly for research purposes.

ABPM: ambulatory blood pressure measurement over 24 h, BP: blood pressure, PTT: pulse transit time, ECG: electrocardiography. The information summarized in Table 1 is based on this work and the references: [2,3,4,5,6].

**Table 2:** Baseline characteristics for all measurements.

Characteristic	Overall (*n* = 234)	
Sex (male), n	148 (63.2%)	
Age, years	51.9 (±13.3, (19–75))	
Calibration systolic BP, mmHg	129 (±23, (81–223))	
Calibration diastolic BP, mmHg	83 (±16, (55–116))	
**Classification**	**Systolic BP**	**Diastolic BP**
Calibration low *, n	130 (55.6)	97 (41.5)
Calibration medium *, n	92 (39.3)	122 (52.1)
Calibration high *, n	12 (5.1)	15 (6.4)
**DominoBP Awake**		
Normotensive, n	144 (61.5)	113 (48.3)
Stage 1 **, n	49 (20.9)	71 (30.3)
Stage 2 **, n	41 (17.5)	50 (21.4)
Severe hypertension ** *°*, n	5 (2.1)	7 (2.9)
**SchillerBP Awake**		
Normotensive, n	142 (60.7)	118 (50.4)
Stage 1 **, *n*	53 (22.6)	66 (28.2)
Stage 2 **, *n*	39 (16.7)	50 (21.4)
Severe hypertension ** *°*, *n*	4 (1.7)	7 (2.9)

Data are mean (± standard deviation or interquartile range, (range)) or counts (n (%)), as appropriate. BP: blood pressure. *: stages according to ESH-IP 2010 protocol: systolic BP low (<130 mmHg), medium (130–160 mmHg), high (>160 mmHg); diastolic BP low (<80 mmHg), medium (80–100 mmHg), high (>100 mmHg) [8].**: stages according to NICE guidelines 2011: systolic BP: normotensive ≤ 135 mmHg, stage 1 ≥ 135 mmHg and < 150 mmHg, stage 2 ≥ 150 mmHg; severe hypertension ≥ 180 mmHg; diastolic BP: normotensive ≤ 85 mmHg, stage 1 ≥ 85 mmHg, stage 2 ≥ 95 mmHg; severe hypertension 3 ≥ 110 mmHg [15]. °: subjects with severe hypertension also included in stage 2 hypertension.

**Table 3:** Electrocardiographic (ECG) characteristics of all patients, and solely patients with > 5 mmHg difference between DominoBP and SchillerBP.

	All Patients n (%) *n* = 234	Patients with ≤ 5 mmHg Difference between DominoBP and SchillerBP *n* = 210 *	Patients with > 5 mmHg Difference between DominoBP and SchillerBP *n* = 24 *	* *p*-Value
Sinus Rhythm	232 (99.1%)	208 (99.0%)	24 (100%)	1.00
Persistent or Paroxysmal Atrial Fibrillation	4 (1.7%)	4 (1.9%)	0 (0%)	1.0
Premature Atrial Contractions ≥ 5%	0 (0%)	0 (0%)	0 (0%)	1.0
Premature Ventricular Contractions ≥ 5%	2 (0.9%)	1 (0.5%)	1 (4.2%)	0.195
Right Bundle Branch Block	7 (3.0%)	5 (2.4%)	2 (8.3%)	0.154
Left Bundle Branch Block	5 (2.1%)	3 (1.4%)	2 (8.3%)	0.0833

*: Fishers exact test two tailed P.

**Table 4:** Comparison of 24-hour, awake, asleep blood pressure values calculated with the DominoBP and SchillerBP software.

BP, mmHg	DominoBP	SchillerBP	*p-*Values
**Systolic**			
24-hour	128 (116–141)	129 (118–142)	0.268
Awake	130 (119–143)	131 (119–144)	0.022
Asleep	126 (114–139)	126 (114–139)	0.424
**Diastolic**			
24-hour	84 (76–92)	84 (76–92)	0.569
Awake	85 (77–93)	85 (77–93)	0.507
Asleep	82 (75–91)	82 (74–91)	0.612

Data are median (IQR), *p*-values are based on Wilcoxon signed-rank tests. BP: blood pressure.

**Table 5:** Mean blood pressure differences and mean absolute blood pressure differences comparing blood pressure values generated by DominoBP and SchillerBP software.

**Systolic BP (mmHg)**	**Mean Difference (SD)**	**Mean Absolute Difference (SD)**
24-hour	0.22 (± 2.59)	1.5 (± 1.7)
Awake	0.39 (± 2.55)	1.5 (± 1.6)
Asleep	−0.04 (± 2.97)	1.7 (± 1.8)
**Diastolic BP (mmHg)**		
24-hour	0.13 (± 2.23)	1.1 (± 1.3)
Awake	0.13 (± 2.23)	1.1 (± 1.5)
Asleep	-0.06 (± 2.71)	1.4 (± 1.9)

BP: blood pressure. SD: standard deviation.

**Table 6:** Agreement between DominoBP and SchillerBP derived blood pressure data for mean systolic blood pressure values in different blood pressure categories, stratified by DominoBP mean systolic awake blood pressure.

	<135 mmHg	≥135; <150 mmHg	≥150 mmHg	≥180 mmHg	Overall
*n* (%)	(*n* = 144)	(*n* = 49)	(*n* = 41)	(*n* = 5)	(*n* = 234)
**24-hour**					
Mean difference, mmHg	0.35 (±2.74)	0.24 (±1.57)	0.03 (±2.87)	−0.20 (±1.64)	0.22 (±2.59)
≤2 mmHg	114 (79.1%)	42 (85.7%)	34 (82.9%)	4 (80%)	190 (81.1%)
≤5 mmHg	136 (94.4%)	49 (100%)	37 (90.2%)	5 (100%)	222 (94.8%)
≤10 mmHg	142 (98.6%)	49 (100%)	40 (97.5%)	5 (100%)	231 (98.7%)
≤15 mmHg	144 (100%)	49 (100%)	41 (100%)	5 (100%)	234 (100%)
**Awake**					
Mean difference, mmHg	0.51 (± 2.65)	0.35 (± 1.82)	0.29 (± 2.81)	−0.20 (±1.64)	0.39 (±2.59)
≤2 mmHg	116 (80.5%)	43 (87.7%)	34 (82.9%)	4 (80%)	193 (82.4%)
≤5 mmHg	139 (96.5%)	48 (97.9%)	38 (92.6%)	5 (100%)	225 (96.1%)
≤10 mmHg	142 (98.6%)	49 (100%)	40 (97.5%)	5 (100%)	231 (98.7%)
≤15 mmHg	144 (100%)	49 (100%)	41 (100%)	5 (100%)	234 (100%)
**Asleep**					
Mean difference, mmHg	0.13 (±3.18)	−0.16 (±1.80)	−0.24 (±3.22)	0.40 (± 2.60)	−0.04 (±2.97)
≤2 mmHg	114 (79.1%)	42 (85.7%)	32 (78.0%)	4 (80%)	188 (80.3%)
≤5 mmHg	135 (93.7%)	48 (97.9%)	36 (87.8%)	5 (100%)	219 (93.5%)
≤10 mmHg	142 (98.6%)	49 (100%)	40 (97.5%)	5 (100%)	231 (98.7%)
≤15 mmHg	143 (99.3%)	49 (100%)	41 (100%)	5 (100%)	233 (99.5%)

Data are mean (± standard deviation) or counts (percent).

**Table 7:** Agreement between DominoBP and SchillerBP derived blood pressure data for mean diastolic blood pressure values in different blood pressure categories, stratified by DominoBP mean diastolic awake blood pressure.

	<85 mmHg	≥85; <95 mmHg	≥95 mmHg	≥110 mmHg	Overall
*n* (%)	(*n* = 113)	(*n* = 71)	(*n* = 50)	(*n* = 7)	(*n* = 234)
**24-hour**					
Mean difference, mmHg	0.22 (± 1.92)	0.35 (± 2.26)	−0.39 (± 2.75)	0.29 (± 0.75)	0.13 (± 2.23)
≤2 mmHg	99 (88.3%)	60 (84.5%)	46 (90.1%)	7 (100%)	205 (87.6%)
≤5 mmHg	111 (99.1%)	68 (95.7%)	49 (96.0%)	7 (100%)	228 (97.4%)
≤10 mmHg	111 (99.1%)	71 (100%)	50 (98.0%)	7 (100%)	232 (99.1%)
≤15 mmHg	112 (100%)	71 (100%)	50 (98.0%)	7 (100%)	233 (99.5%)
**Awake**					
Mean difference, mmHg	0.23 (± 1.93)	0.34 (± 2.24)	−0.39 (± 2.75)	−0.14 (± 0.90)	0.13 (± 2.23)
≤2 mmHg	100 (79.1%)	62 (87.3%)	46 (90.1%)	7 (100%)	208 (88.8%)
≤5 mmHg	111 (99.1%)	68 (95.7%)	49 (96.0%)	7 (100%)	228 (93.5%)
≤10 mmHg	111 (99.1%)	70 (98.5%)	50 (98.0%)	7 (100%)	231 (98.2%)
≤15 mmHg	112 (100%)	71 (100%)	50 (98.0%)	7 (100%)	233 (99.5%)
**Asleep**					
Mean difference, mmHg	0.04 (± 2.54)	0.18 (± 2.45)	−0.61 (± 3.34)	0.00 (± 0.57)	−0.06 (± 2.71)
≤2 mmHg	99 (88.3%)	61 (85.9%)	44 (86.2%)	7 (100%)	204 (87.1%)
≤5 mmHg	107 (95.5%)	68 (95.7%)	48 (94.1%)	7 (100%)	223 (95.2%)
≤ 10 mmHg	110 (98.2%)	71 (100%)	49 (96.0%)	7 (100%)	230 (98.2%)
≤15 mmHg	112 (100%)	71 (100%)	50 (98.0%)	7 (100%)	233 (99.5%)

Data are mean (± standard deviation) or counts (percent).

**Table 8:** Baseline characteristics for patients with an absolute systolic or diastolic blood pressure difference of > 5 mmHg between data generated by DominoBP and SchillerBP software in 24-h, awake, or asleep blood pressure measurement.

Characteristics	Overall (*n* = 24)	
Sex (male), n	18 (75%)	
Age, years	54.5 (±13.1, (31–74))	
Calibration systolic BP, mmHg	132.8 (±30.3, (98–223))	
Calibration diastolic BP, mmHg	83 (±13.8, (62–112))	
	Systolic BP	Diastolic BP
Absolute BP Differences > 5mmHg n (% of 234 patients)		
24-hour	12 (5.1%)	6 (2.5%)
Awake	9 (3.8%)	6 (2.5%)
Asleep	15 (6.4%)	11 (4.7%)
Blood Pressure Ranges in Different BP Categories n/subjects (%)		
Calibration BP		
Low *	13/130 (10%)	11/97 (11.3%)
*Medium*	9/92 (9.8%)	12/122 (9.8%)
*High*	2/12 (16.7%)	1/1 (6.7%)
DominoBP Awake		
*Normotensive*	14/144 (9.7%)	11/113 (9.7%)
Stage 1 **	4/49 (8.2%)	7/71 (9.9%)
Stage 2 **	6/41 (14.6%)	6/50 (12%)
Severe hypertension ** °	1/5 (20%)	0/7 (0%)
SchillerBP Awake		
*Normotensive*	11/142 (7.7%)	14/11 (11.9%)
Stage 1 **	8/53 (15.1%)	3/66 (4.5%)
Stage 2 **	5/39 (12.8%)	7/50 (14%)
Severe hypertension ** °	1/4 (25%)	1/7 (14.3%)

Data are mean (± standard deviation or interquartile range, (range)) or counts (female), as appropriate. BP: blood pressure. *: stages according to ESH-IP 2010 protocol: systolic BP low (<130 mmHg), medium (130–160 mmHg), high (> 160 mmHg); diastolic BP low (<80 mmHg), medium (80–100 mmHg), high (>100 mmHg) [8]; **: stages according to the NICE guidelines 2011: systolic BP: normotensive ≤ 135 mmHg, stage 1 ≥ 135 mmHg and < 150 mmHg, stage 2 ≥ 150 mmHg, severe hypertension ≥ 180 mmHg; diastolic BP: normotensive ≤ 85 mmHg, stage 1 ≥ 85 mmHg, stage 2 ≥ 95 mmHg, severe hypertension 3 ≥ 110 mmHg [15]; °: subjects with severe hypertension also included in stage 2 hypertension.
